# Ruxolitinib suppresses liver fibrosis progression and accelerates fibrosis reversal via selectively targeting Janus kinase 1/2

**DOI:** 10.1186/s12967-022-03366-y

**Published:** 2022-04-05

**Authors:** Zhenghui Song, Xinhui Liu, Wan Zhang, Yue Luo, Hua Xiao, Yun Liu, Guanqi Dai, Jian Hong, Aimin Li

**Affiliations:** 1 Department of Hepatology, Cancer Center, Integrated Hospital of Traditional Chinese Medicine, Southern Medical University, No.13 Shiliugang Road, Guangzhou, 510315 Guangdong China; 2 School of Traditional Chinese Medicine, Southern Medical University, Guangzhou, 510515 Guangdong China; 3 Department of Physiology, Michigan State University, East Lansing, MI 48824 USA; 4 Department of Endocrinology and Metabolic Diseases, Affiliated Hospital (Clinical College) of Xiangnan University, Chenzhou, 423000 China; 5 School of Medicine, Jinan University, Guangzhou, 510632 China

**Keywords:** Janus Kinase 1, Janus Kinase 2, Liver Fibrosis, HSCs, Ruxolitinib

## Abstract

**Background:**

JAK1 and JAK2 have been implicated in fibrosis and cancer as a fibroblast-related marker; however, their role in liver fibrosis has not been elucidated. Here, we aim to determine the effect and underlying mechanism of JAK1/2 inhibition on liver fibrosis and hepatic stellate cells (HSCs) and further explore the therapeutic efficacy of Ruxolitinib, a JAK1/2 selective inhibitor, on preventing and reversing liver fibrosis in mice.

**Methods:**

Immunohistochemistry staining of JAK1 and JAK2 were performed on liver tissue in mice with hepatic fibrosis and human liver tissue microarray of liver cirrhosis and liver cancer. LX-2 cells treated with specific siRNA of JAK1 and JAK2 were used to analysis activation, proliferation and migration of HSCs regulated by JAK1/2. The effects of Ruxolitinib (JAK1/2 inhibitor) on liver fibrosis were studied in LX-2 cells and two progressive and reversible fibrosis animal models (carbon tetrachloride (CCl_4_), Thioacetamide (TAA)).

**Results:**

We found that JAK1/2 expression was positively correlated with the progression of HCC in humans and the levels of liver fibrosis in mice. Silencing of JAK1/2 down-regulated their downstream signaling and inhibited proliferation, migration, and activation of HSCs in vitro, while Ruxolitinib had similar effects on HSCs. Importantly, Ruxolitinib significantly attenuated fibrosis progression, improved cell damage, and accelerated fibrosis reversal in the liver of mice treated with CCl_4_ or TAA.

**Conclusions:**

JAK1/2 regulates the function of HSCs and plays an essential role in liver fibrosis and HCC development. Its inhibitor, Ruxolitinib, may be an effective drug for preventing and treating liver fibrosis.

**Supplementary Information:**

The online version contains supplementary material available at 10.1186/s12967-022-03366-y.

## Background

Liver fibrosis is a chronic liver injury mainly caused by hepatitis virus B and C chronic infection, excess alcohol consumption, nonalcoholic steatohepatitis (NASH), autoimmune liver diseases and hereditary diseases.[1]. Liver fibrosis eventually progresses to liver cirrhosis, a common cause of death worldwide [2], resulting in serious complications, including portal hypertension, liver failure, and hepatocellular carcinoma (HCC), leading to failure of liver function and destruction of liver structures, and ultimately organ dysfunction and death [3]. Currently, liver transplantation is the only effective cure, which brings a tremendous economic burden to the patients [4]. Liver fibrosis is due to excessive accumulation of extracellular matrix (ECM) in response to chronic liver damage. Hepatic stellate cells (HSCs) are considered the most critical cell type for the production of collagens [5–7]. When stimulated by liver damage, HSCs will be activated, and then differentiate into myofibroblasts acquire fibrogenic property for producing ECM, resulting in fibrotic scar [8, 9]. Many drug companies try to find methods to halting scarring or even remove existing scars, but no drug is yet approved for treating cirrhosis [10]. Thus, the development of effective anti-fibrotic drugs to stop progression to cirrhosis or even reverse advanced fibrosis is urgently needed [11].

Several signaling pathways are found to be related to liver fibrosis, and may be potential targets for treatment. The Janus kinase/Signal Transducer and Activator of Transcription protein (JAK/STAT) signaling pathway is a chain of interactions between proteins and involved in processes such as immunity, cell division, cell death, and tumor formation [12–16]. Recent studies have reported that STATs are associated with tissue fibrosis, including skin, lung, liver, systemic sclerosis (SSc), and STAT3 inhibitors have been shown to be effective in the Carbon tetrachloride (CCl_4_)-induced liver fibrosis model [17, 18]. JAKs act as the upstream of STATs, and JAK/STAT signaling pathway plays a role in promoting the fibrosis, and selective JAK2 inhibitor can effectively block fibroblast activation improve fibrosis [19]. Besides, recent studies suggested that JAK1 might also play an indirect role in promoting fibrosis through the transphosphorylation of JAK2 [20]. However, the role of JAK1 and JAK2 in liver fibrosis remains unclear.

Ruxolitinib is the only effective small-molecule JAK1/2 selective inhibitor approved by the US Food and Drug Administration (FDA) for myelofibrosis treatment in 2011. It is mainly used for the treatment of intermediate or high-risk bone marrow fibers, which is well tolerated in a multicenter study and can effectively alleviate splenomegaly, improve spleen hyperfunction and systemic symptoms of patients [21–23]. Recently, it has been reported that Ruxolitinib is effective and safe for the treatment of hematological diseases such as polycythemia vera, lymphoma, etc.[24, 25], and can benefit some patients with pancreatic cancer and breast cancer [25, 26]. Currently, a series of clinical studies of Ruxolitinib in malignant glioma, multiple myeloma, lung cancer, breast cancer, colorectal cancer, head, and neck squamous cell carcinoma, and prostate cancer are underway (https://clinicaltrials.gov/ct2/results?cond=ruxolitinib). However, the efficacy of Ruxolitinib on liver fibrosis and liver cancer has not been explored.

In the present study, we found up-regulated JAK1 and JAK2 were associated with liver disease in humans and mice, while knockdown of JAK1 and JAK2 inhibited activation, proliferation, and migration of HSCs. Also, Ruxolitinib, the JAK1/2 selective inhibitor, blocked HSCs activation, both in cell culture and in animal fibrosis models, included by CCl_4_ and Thioacetamide (TAA). Furthermore, we found that Ruxolitinib attenuated liver fibrosis progression, accelerated its reversal, and protected from liver damage, which provides a new direction and clinical transformation basis for the treatment of liver fibrosis and even early intervention of HCC.

## Methods

### Cell culture

Immortalized human HSC cell line (LX-2) was purchased from Chinese Academy of Sciences Cell Bank (Shanghai, China) and cultured in Dulbecco’s modified Eagle’s medium (DMEM) (Gibco, China), supplemented with 10% fetal bovine serum (FBS) (Gibco, USA) and 1% Penicillin/Streptomycin at 37 °C humidified chamber with 5% CO_2_. All cell experiments were performed in strict accordance with cell culture protocols.

### Chemicals

For in vitro experiments, Ruxolitinib (INCB018424, Selleck chemicals, USA) was dissolved in DMSO and further diluted to the required concentration. For in vivo experiments, Ruxolitinib suspension was prepared in 0.5% carboxymethyl cellulose sodium normal saline solution.

### Small interfering RNA (siRNA) Transfection

The specific siRNA for JAK1 and JAK2 were designed and synthesized by RiboBio (Guangzhou, China). The siRNA sequences were siJAK1: CCACATAGCTGATCTGAAA; siJAK2: ATGACTTTGTCATGTCTTA. siRNAs were transfected into cells using Lipofectamine 3000 (Invitrogen) according to the manufacturer’s protocol. Cells were collected after 48–72 h for further experiments.

### Human samples

Human liver tissue microarray was purchased from Biomax, including 5 liver samples with normal liver tissue, 22 liver samples with liver cirrhosis, and 53 liver samples with liver cancer which were collected in US.

### Histology and immunohistochemistry

Formalin-fixed, paraffin-embedded liver tissue samples were cut into 4 µm-thick sections and stained with hematoxylin eosin (H&E), Sirius red, and immunohistochemistry (IHC) according to standard procedures. Fibrosis was scored according to the Ishak scoring system [27]. The amount of Sirius red staining was quantified with ImageJ. For IHC, liver sections were stained with the following antibodies: JAK1 (sc-1677; Santa Cruz Biotechnology), JAK2 (#3230; Cell signaling Technology), Alpha-Smooth muscle actin (α-SMA,1A4, ab7817; Abcam), Both the intensity and extent of immunostaining were taken into consideration when analyzing the data. The intensity of staining was determined by the following rules: 0 for negative; 1 for weak staining; 2 for moderate staining; 3 for strong staining. The staining extent was determined by the following rules: 0 for no staining; 1 for less than 10%; 2 for 10% to 50%; 3 for 51% to 75%; 4 for more than 75%. We randomly selected 5 areas from each area to count the intensity and extent of staining and to calculate the mean staining extent. The score was obtained by plus these two values (intensity score + extent score).

### Liver function assay

Serum levels of several biochemical markers were measured to assess liver function and liver injury. The measured biochemical markers included alkaline phosphatase (ALP), alanine aminotransferase (ALT), aspartate aminotransferase (AST), total bilirubin (TBIL) and albumin (Alb) were measured using standard laboratory assays [2].

### Western blotting

Western blotting was performed as previously described [28]. The primary antibodies were purchased from Santa Cruz Biotechnology [JAK1 (sc-1677)], Cell signaling Technology [JAK2 (#3230), phosho-JAK1 (Tyr1022/1023, #3331), phosho-JAK2 (Tyr1007/1008, #3771), STAT3(#9139), phosho-STAT3 (Tyr705,#9145), phosho-STAT3 (Ser705, #9134), STAT5 (#94205), phosho-STAT5 (Tyr694, #9359)], Abcam [α-SMA (1A4, ab7817)], Bimake [PDGFRβ (A5541), PAI-1 (A5396)], Bioworld [β-actin (#64132)]. β-actin was used as a loading control for all blots.

### Quantitative Real-time RT-PCR

Total RNA was extracted from cells and tissues using Trizol (Takara, Japan), and was reverse transcribed into cDNA according to the manufacturer’s instructions, and the GAPDH gene was used as gene control. The relative gene expression ratio was calculated by the ΔΔCt method. The specific primers were listed as follows: JAK1: 5’-CCACTACCGGATGAGGTTCTA-3’ (forward) and 5’-GGGTCTCGAATAGGAGCCAG-3’ (reverse); JAK2: 5’-GCCGGGTTTCAGAAGCAGG-3’ (forward) and 5’- GTAAGGCAGGCCATTCCCAT -3’ (reverse); ACTA2: 5’-GACAATGGCTCTGGGCTCTGTAA -3’ (forward) and 5’-CTGTGCTTCGTCACCCACGTA-3’ (reverse); PAI-1: 5’- AGTGGACTTTTCAGAGGTGGA-3’ (forward) and 5’- GCCGTTGAAGTAGAGGGCATT -3’ (reverse); PDGFRβ: 5’-GCCCTTATGTCGGAGCTGAAGA-3’ (forward) and 5’-GTTGCGGTGCAGGTAGTCCA-3’ (reverse); COL1A1: 5’-CAGCCGCTTCACCTACAGC-3’ (forward) and 5’-TCAATCACTGTCTTGCCCCA-3’ (reverse); GAPDH: 5’-TGTTGCCATCAATGACCCCTT -3’ (forward) and 5’-CTCCACGACGTACTCAGCG-3’ (reverse). Experiments were performed according to the manufacturer’s instructions (Takara, Japan). Independent experiments were done in triplicate.

### Cell proliferation assay

LX-2 cells were seeded at a density of 3000 cells/well in 96-well microplates. The cells were treated with siRNA transfection or varying concentrations of Ruxolitinib as designed (0.1–100 μM). Cell viability was measured using the Cell Counting Assay Kit-8 (CCK-8; Dojindo, Japan) according to the manufacturer’s instructions. For each experimental condition, five parallel wells were assigned to each group. Experiments were performed in triplicate.

### Cell migration assay

LX-2 cells were seeded in a 6-well plate treated with siRNA transfection or drug for 24 h. Migration assays were conducted using 24-well Boyden chambers containing inserts (8 μm pores; BD Biosciences,USA). The lower chamber was filled with medium containing 10% serum, whereas the top chamber contained 1 × 10^5^ cells without serum. The plates were incubated at 37 °C in 5% CO_2_ for 10 h. After migration, the cells that had migrated to the underside of the membrane were fixed with paraformaldehyde and stained with 0.1% crystal violet. Migrated cells on each insert were counted in five randomly selected fields and quantified using the ImageJ software.

### Cell apoptosis assay

LX-2 cells were seeded in 6-well plates and treated with different concentrations of Ruxolitinib for 24 h. For cells were then harvested and stained with Annexin V-FITC Apoptosis Detection Kit (BD Pharmingen, CA) according to the manufacture’s protocol. FACS caliber flow cytometry (BD Biosciences, USA) was used to assess apoptotic rate. The sum of early and late apoptotic cells was measured.

### Mouse models

All mice were housed in the Animal Facility at Southern Medical University under standard pathogen-free conditions, and were maintained at 16–27℃, 30–70% humidity and a 12-h light/dark cycle. All animal experiments were conducted in accordance with the National Institutes of Health guide for the care and use of Laboratory animals s and approved by the Animal Care and Use Committee of Southern Medical University. Each treatment group comprised 6–10 mice (N = 6–10).

### Liver fibrosis progression and reversal model induced by CCl_4_

For the Liver fibrosis progression model, 4–6 weeks old male C57BL/6 mice (Vital River, Beijing, China) were treated three times a week with or without 0.1 ml of a 40% CCl_4_ in olive oil by oral gavage for 8 weeks and mice were treated with or without Ruxolitinib (30 mg/kg, oral gavage, each day) from 5 to 8 weeks. For Liver fibrosis reversal model, 4–6 weeks old male C57BL/6 mice (Vital River, Beijing, China) were treated three times a week with or without 0.1 ml of a 40% CCl_4_ in olive oil by oral gavage for 6 weeks, and then mice allowed to recover from 6 to 8 weeks, with or without treatment with Ruxoltinib (30 mg/kg, oral gavage, each day).

### Panlobular liver fibrosis model induced by TAA

6–8 weeks old male C57BL/6 mice were accepted an optimistic dose-escalating TAA as described [29]. And then mice were allowed to recover from 6 to 10 weeks, with or without treatment with Ruxolitinib (30 mg/kg, oral gavage, each day).

### Statistical analysis

All statistical analyses were performed with GraphPad Prism V.5.00. Data are expressed as mean ± standard deviation (SD). Differences between two groups were compared using a two-tailed unpaired Student’s t-test. One-way ANOVA was used for sample comparison among multiple groups. Statistical significance was defined as *P* < 0.05.

## Results

### Up-regulated JAK1 and JAK2 are associated with liver fibrosis and liver disease in human and mice

**Fig.1:**
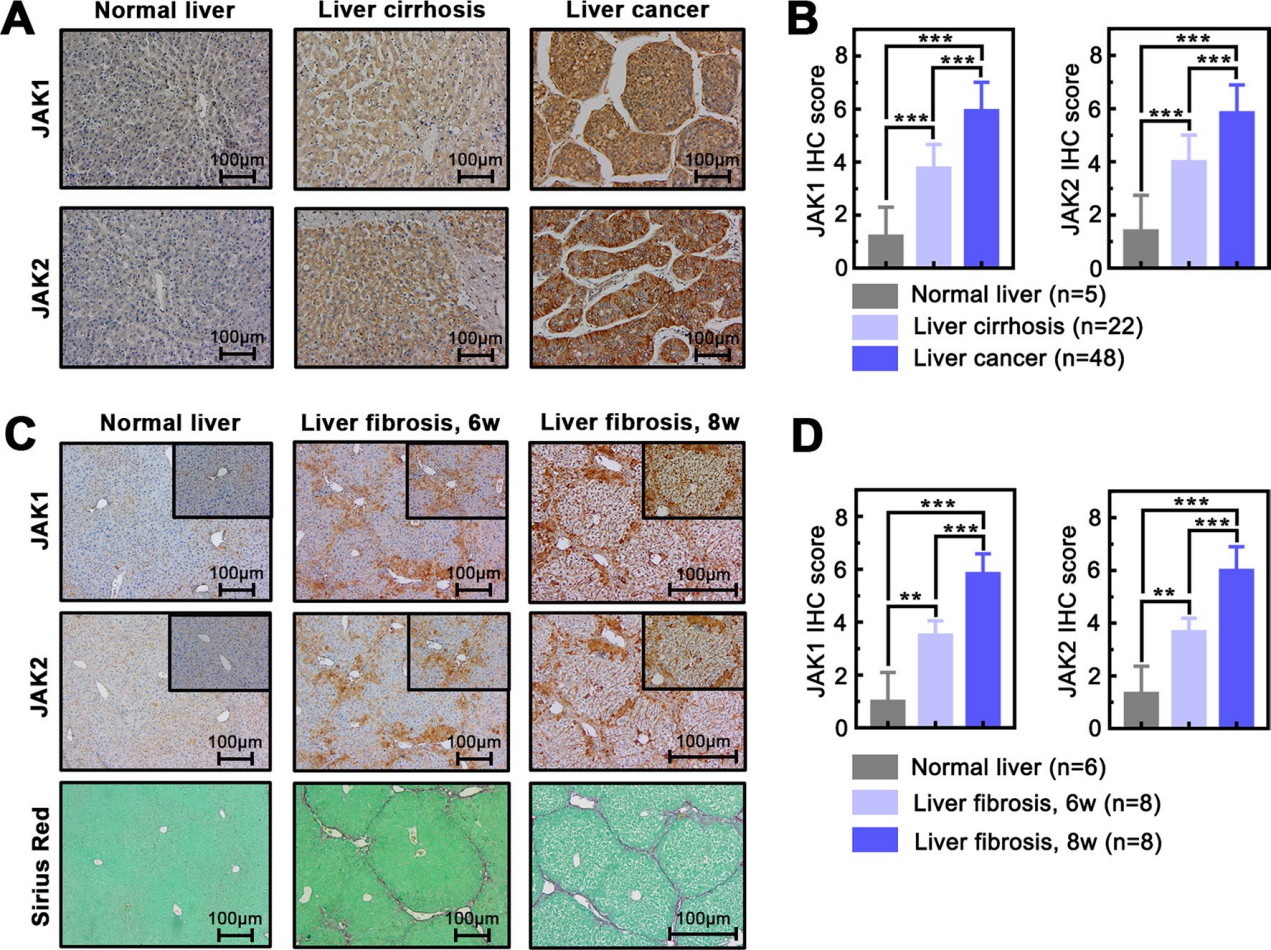
Up-regulated JAK1 and JAK2 are associated with liver fibrosis and liver disease in human and mice. **A** JAK1 and JAK2 immunohistochemistry (IHC) was performed in human liver tissues microarray of normal, cirrhosis, and cancer (× 100 magnification, scale bar = 100 μm). **B** JAK1 and JAK2 scores quantified by IHC in normal liver, liver cirrhosis, and liver cancer. **C** The IHC and Sirius red staining of JAK1 and JAK2 were performed in mouse normal and fibrosis for 6 weeks CCl_4_-induced or 8 weeks CCl_4_-induced liver tissues (× 100 and × 200 magnification, scale bar = 100 μm). **D** JAK1 and JAK2 scores quantified by IHC in normal liver, 6 weeks CCl_4_-induced liver fibrosis and 8 weeks CCl_4_-induced liver fibrosis. Data presented are means ± SD. **P < 0.01; ***P < 0.001

To evaluate whether JAK1 and JAK2 expression are associated with liver fibrosis/cirrhosis and liver cancer, IHC staining was used to examine JAK1 and JAK2 expression in human liver tissue array with confirmed cirrhosis and cancer. JAK1 and JAK2 expression were markedly higher in liver cirrhosis tissues (n = 22), while highest in liver cancer tissues (n = 53) compared to normal liver tissues (n = 5) (Fig. 1A, B). We further analyzed the relationship between the expression of JAK1 and JAK2 and the degree of liver fibrosis in mice. Sirius Red staining was used to confirm constructed liver fibrosis mouse model induced by CCl_4_ for 6 or 8 weeks respectively, and IHC staining showed expression of JAK1 and JAK2 were a significant increase in fibrosis liver compared with controls (Fig. 1C, D, which is constant to human liver disease. Our results suggest that the expression of JAK1 and JAK2 was associated with liver cancer progression and was also positively correlated with the severity of liver fibrosis.

### Knockdown of JAK1 and JAK2 inhibits activation, proliferation and migration of HSCs

**Fig.2:**
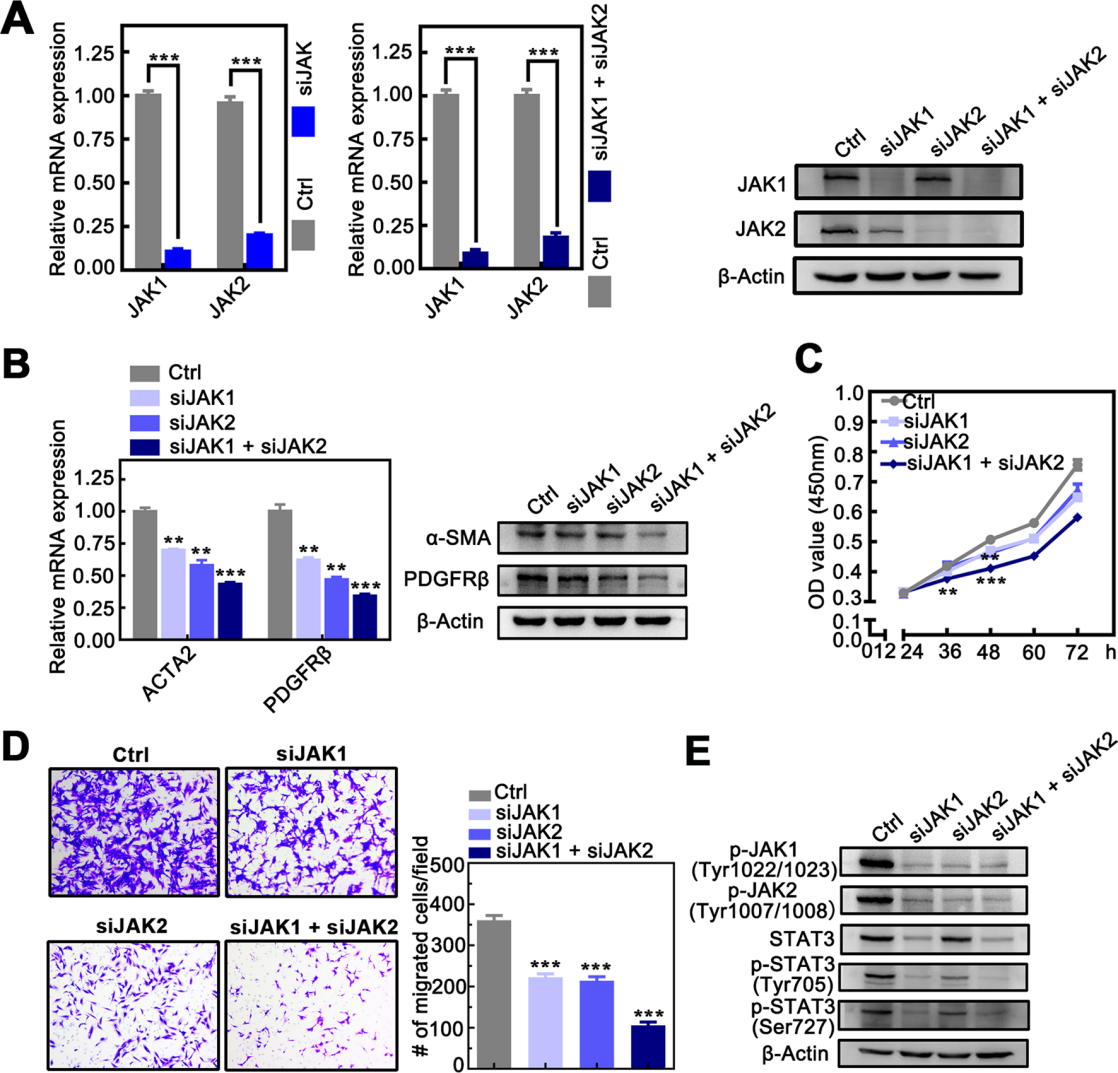
Knockdown of JAK1 and JAK2 inhibits activation, proliferation and migration of HSCs. **A** The left panel shows LX-2 cells co-interfered with siJAK1 and siJAK2 reduced JAK1 and JAK2 mRNA expression. The right panel shows that siJAK2 reduced JAK1 and JAK2 protein expression in LX-2 cells co-interfered with siJAK1. **B** The left panel shows that knockdown JAK1 and JAK2 reduced mRNA expression of ACTA2, PDGFRβ in LX-2 cells. The right panel shows knockdown JAK1 and JAK2 reduced protein expression of α-SMA, PDGFRβ. **C** Knockdown expression of JAK1 and JAK2 inhibited LX-2 cell proliferation. **D** The left panel shows knockdown JAK1 and JAK2 inhibited migration (× 100 magnification). The right shows the number of migrations counted after interfered with JAK1 and JAK2 in LX-2 cells. **E** LX-2 cells knockdown JAK1 and JAK2 reduced protein expression of p-JAK1 (Tyr1022/1023), p-JAK2 (Tyr1007/1008), STAT3, p-STAT3 (Tyr705), p-STAT3 (Ser727). Date presented are means ± SD. *P < 0.05; **P < 0.01; ***P < 0.001

Since the activation and proliferation of HSCs in the main trigger for liver fibrosis and subsequent liver disease, to investigate the role of JAK1 and JAK2 on HSCs, LX-2 cells were transfected with siJAK1, siJAK2, or both of them. The transfected efficiency was confirmed by qRT-PCR and Western blotting (Fig. 2A). As α-SMA and platelet-derived growth factor receptor beta (PDGFRβ) is the common marker for HSCs activation, the mRNA and protein level were detected to investigate the role of JAK1 and JAK2 on the activation of HSCs. As shown in Fig. 2B, α-SMA and PDGFRβ were significantly decreased in LX-2 cells transfected with siJAK1 or siJAK2. Furthermore, the lowest expression was found in cells co-transfected with siJAK1 and siJAK2.

Next, we analyzed the effect of JAK1 and JAK2 on the proliferation and migration of the HSCs. Knockdown of JAK1 or JAK2 had a similar impact on inhibiting proliferation, while the proliferation of HSCs was further decreased in the co-transfected group (Fig. 2C). Likewise, the number of migrated HSCs was significantly reduced in cells with siJAK1 or siJAK2 and lowest in the co-transfected group compared to the control group (Fig. 2D). Furthermore, we investigated the effect of JAK1 and JAK2 on the downstream signaling and revealed that expression of p-JAK1, p-JAK2, STAT3, and p-STAT3 was significantly decreased (Fig. 2E). These results indicate that knockdown JAK1 and JAK2 may inhibit the activation, proliferation and migration of HSCs.

### JAK1 and JAK2 antagonism has anti-fibrotic activity in vitro

**Fig.3:**
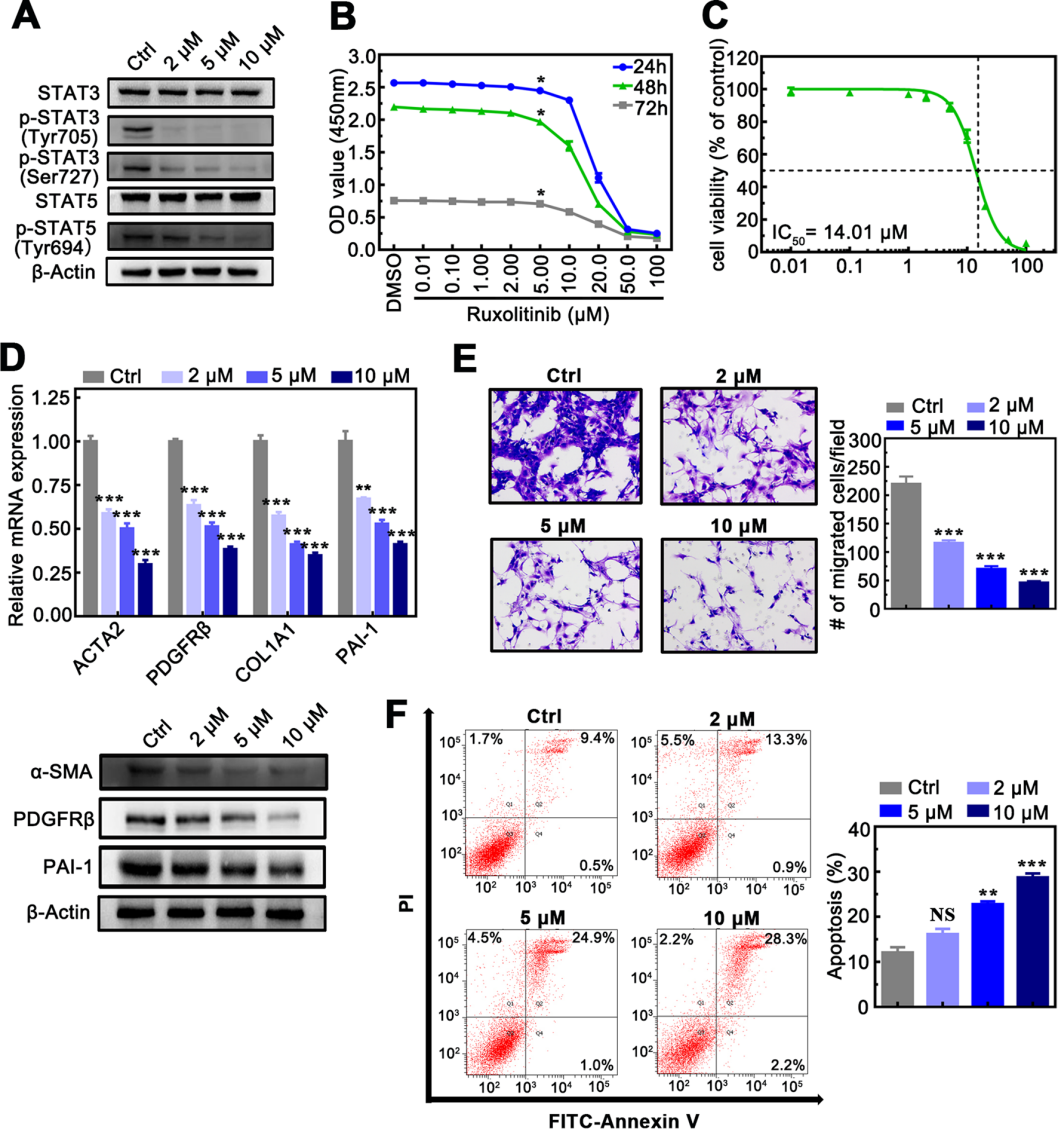
JAK1 and JAK2 antagonism had anti-fibrotic activity in vitro. **A** The protein levels of JAK downstream in LX-2 cells treated with different concentrations of Ruxolitinib. **B** Ruxolitinib inhibited cell proliferative effect in LX-2 cells in a dose and time-dependent manner. **C** The half-maximal inhibitory concentration (IC_50_) value of Ruxolitinib on LX-2 cells. **D** The upper panel shows relative mRNA expression of ACTA2, PDGFRβ, COL1A1, and PAI-1 in LX-2 cells treated with different concentrations of Ruxolitinib. The below shows protein expression of α-SMA, PDGFRβ, and PAI-1. **E** The left panel shows that treatment with Ruxolitinib inhibited LX-2 cell proliferation (× 100 magnification). The right panel shows the number of migration counted in LX-2 cells treated with different concentrations of Ruxolitinib. **F** The left panel shows that treatment with Ruxolitinib promoted LX-2 cells apoptosis. The right panel shows the apoptosis rate of LX-2 cells treated with different concentrations of Ruxolitinib. Data presented are means ± SD. *NS* not significant; *P < 0.05; **P < 0.01; ***P < 0.001

Ruxolitinib is a selective small-molecule oral inhibitor of JAK1/2, and it was verified to effectively inhibit JAK1 phosphorylation at Tyr1022/1023 and JAK2 phosphorylation at Tyr1007/1008, the critical activation site at the kinase domain of JAK1 and JAK2, respectively (Additional file 1: Fig.S1). Moreover, we revealed that Ruxolitinib significantly decreased STAT3 phosphorylation at Tyr705 and Ser727, STAT5 phosphorylation at Tyr694, while no significant changes were observed in STAT3 and STAT5 (Fig. 3A). Then we utilized Ruxolitinib to investigate whether JAK1 and JAK2 antagonism has anti-fibrotic activity.

By using Ruxolitinib on LX-2 cells, we found Ruxolitinib inhibits cell proliferation in a dose and time-dependent manner in LX-2 cells (Fig. 3B), and the half- maximal inhibitory concentration (IC_50_) of Ruxolitinib on LX-2 cell was 14.01 μM (Fig. 3C). Furthermore, Ruxolitinib significantly inhibited the expression of HSCs activation marker (*α-SMA* and *PDGFRβ*) and collagen-associated marker (*COL1A1* and *PAI-1*) (Fig. 3D). Besides, Ruxolitinib also significantly inhibited the number of migrations (Fig. 3E) and induced the level of apoptosis (Fig. 3F) in LX-2 cells in a dose-dependent manner. These results demonstrate that Ruxolitinib, as the JAK1 and JAK2 antagonism, inhibits proliferation, activation, migration and promoted cell apoptosis of HSCs in vitro and may exert an anti-fibrotic activity by inhibiting phosphorylation site of JAK1/2 downstream signaling.

### Ruxolitinib attenuates the progression of liver pan-lobular fibrosis induced by CCl_4_

To determine the appropriate dose of Ruxolitinib in vivo, we explored and conformed the appropriate drug concentration (30 mg/kg) in the preliminary experiment and verified it had no side effects on normal mice, which was used in the subsequent animal experiments (Additional file 1: Fig.S2).

**Fig.4:**
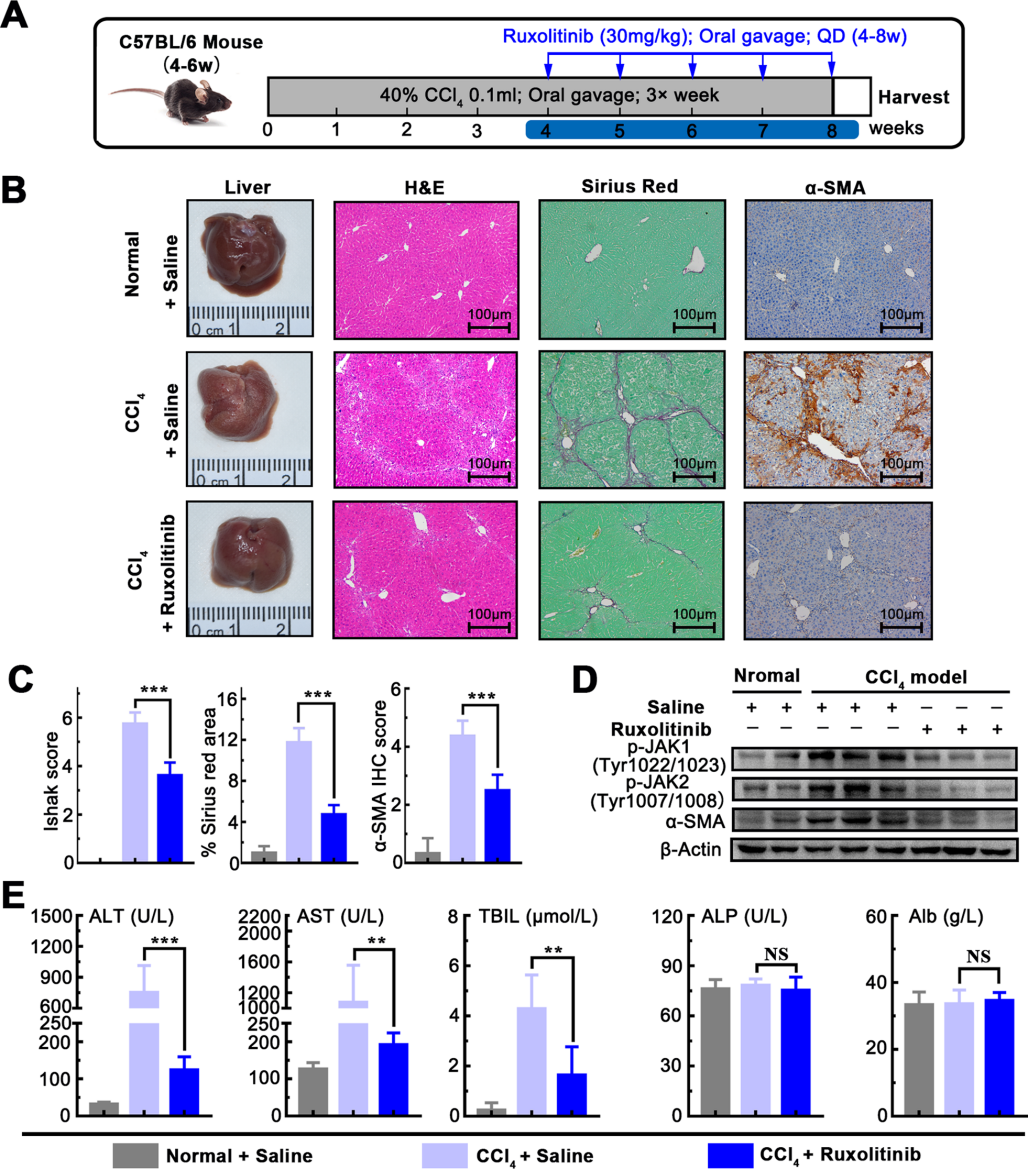
Ruxolitinib attenuates the progression of liver pan-lobular fibrosis induced by CCl_4_. **A** Schematic of the experimental design of Ruxolitinib treatment in a CCl_4_-induced fibrosis progression model in mice. **B** Representative images of mouse livers stained with H&E, Sirius red, and α-SMA antibodies. **C** Ruxolitinib reduced Ishak fibrosis score, and Sirius red, α-SMA IHC staining (× 100 magnification, scale bar = 100 μm). **D** Ruxolitinib reduced the protein expression of p-JAK1, p-JAK2, and α-SMA. **E** Serum levels of ALP, ALT, TBIL, ALP and Alb. Data presented are means ± SD. *NS* not significant; **P < 0.01; ***P < 0.001

Progressive liver pan-lobular fibrosis was induced in C57BL/6 mice by repeated CCl_4_ oral gavage for up to 8 weeks, as described above. To evaluate the anti-fibrotic efficacy of Ruxolitinib on advanced liver fibrosis, we administered Ruxolitinib (30 mg/kg, oral gavage, each day) concurrently with fibrosis induction, from 4 to 8 weeks while vehicle control were administered in parallel (Fig. 4A). Gross morphology and HE staining analysis revealed that Ruxolitinib significantly reduced liver fibrosis and further confirmed by Sirius red staining. Also, IHC staining showed expression of α-SMA was increased in mice with CCl_4_ while significantly decreased in mice treated with Ruxolitinib highlighting the inhibition of Ruxolitinib on the activation of HSCs (Fig. 4B, C). Moreover, Western blotting showed that Ruxolitinib effectively inhibits the expression of p-JAK1, p-JAK2, and α-SMA (Fig. 4D). To explore that the effect of Ruxolitinib on hepatic function, serum level of ALT, AST, TBIL, ALP, and Alb were examined. We found that Ruxolitinib treatment partially decreased the level of ALT, AST, and TBIL, which is increased by CCl_4_, indicating that it may partially protect the liver from damage (Fig. 4E). These results suggest that Ruxolitinib attenuates CCl_4_-induced liver fibrosis by targeting JAK1/2 and activation of HSCs.

### Ruxolitinib accelerates the reversal of liver fibrosis in mice

**Fig.5:**
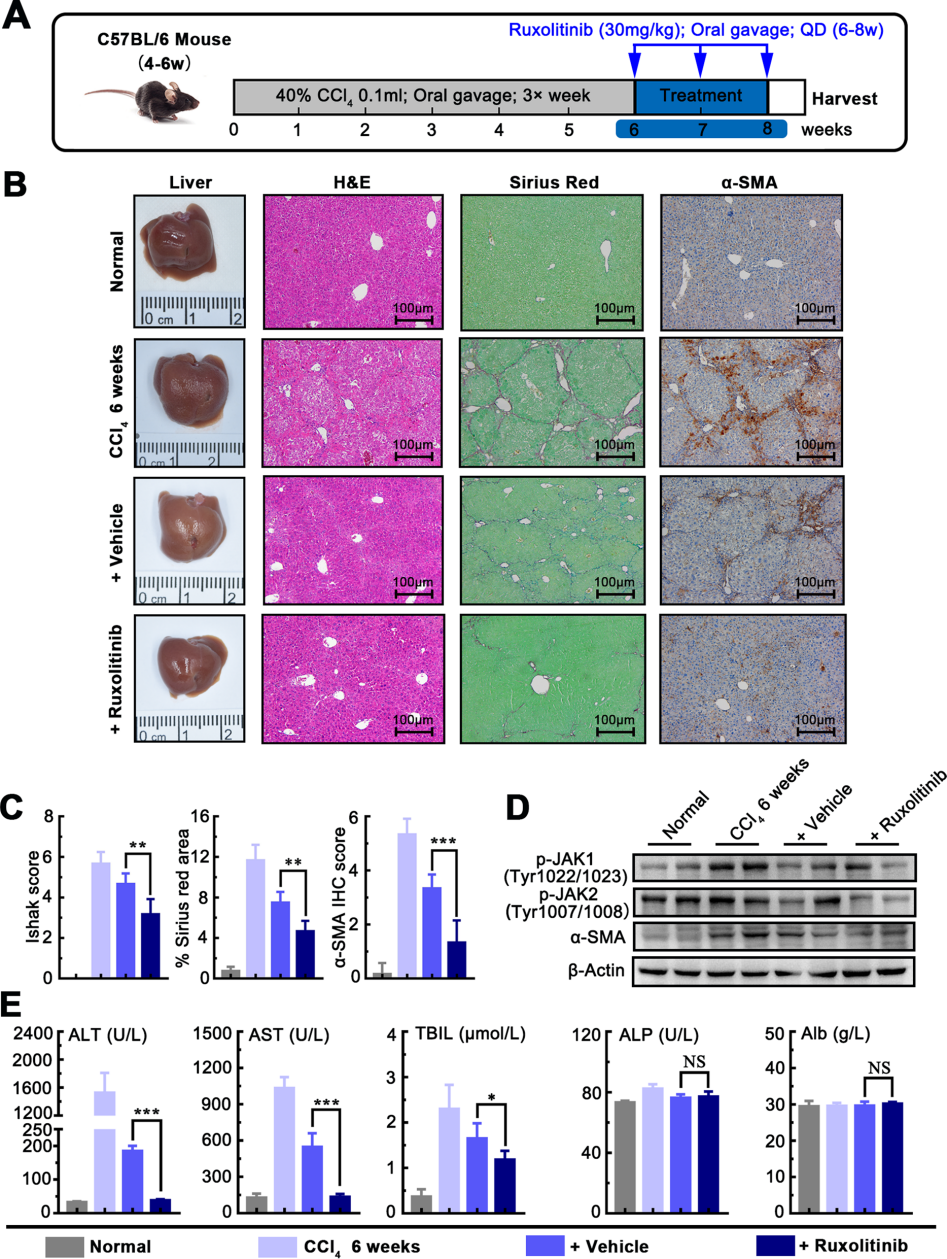
Ruxolitinib accelerates the reversal of liver fibrosis in CCl_4_ mouse model. **A** Schematic of the experimental design of Ruxolitinib treatment in a CCl_4_-induced fibrosis reversal model in mice. **B** Representative images of mouse livers stained with H&E, Sirius red and α-SMA antibodies. **C** Ruxolitinib reduced Ishak fibrosis score, and Sirius red, α-SMA IHC staining (× 100 magnification, scale bar = 100 μm). **D** Ruxolitinib reduced the protein expression of p-JAK1, p-JAK2, and α-SMA. **E** Serum levels of ALP, ALT, TBIL, ALP, and Alb. Date presented are means ± SD. *NS* not significant; *P < 0.05; **P < 0.01; ***P < 0.001

Since exploring effective intervention for reversing liver fibrosis in meaning for liver disease, we sought to test whether Ruxolitinib may promote liver fibrosis reversal in different mice models induced by CCl_4_ or TAA, which is widely used as a reversible model for study drug intervention [29]. As shown in Fig. 5A, the liver fibrosis mice were induced by CCl_4_ for 6 weeks, and then mice were allowed to recover from 6 to 8 weeks, with or without treatment with Ruxolitinib. Morphological analysis and tissue staining showed that the development of liver fibrosis and activation of HSCs in the mice treated with CCl_4_ for 6 weeks, while partially spontaneous reversion was found in 8-week-old mice without Ruxolitinib. Interestingly, the degree of liver fibrosis and the activation of HSCs were significantly reversed in 8-week-old mice treated with Ruxolitinib for 2 weeks which is confirmed by western blot (Fig. 5B–D). Furthermore, the serum markers of hepatic function were examined, and as shown in Fig. 5E, the levels of ALT, AST, and TBIL were significantly reduced in mice with Ruxolitinib compared that those without Ruxolitinib, indicating Ruxolitinib may promote the improvement of liver function in mice.

**Fig.6:**
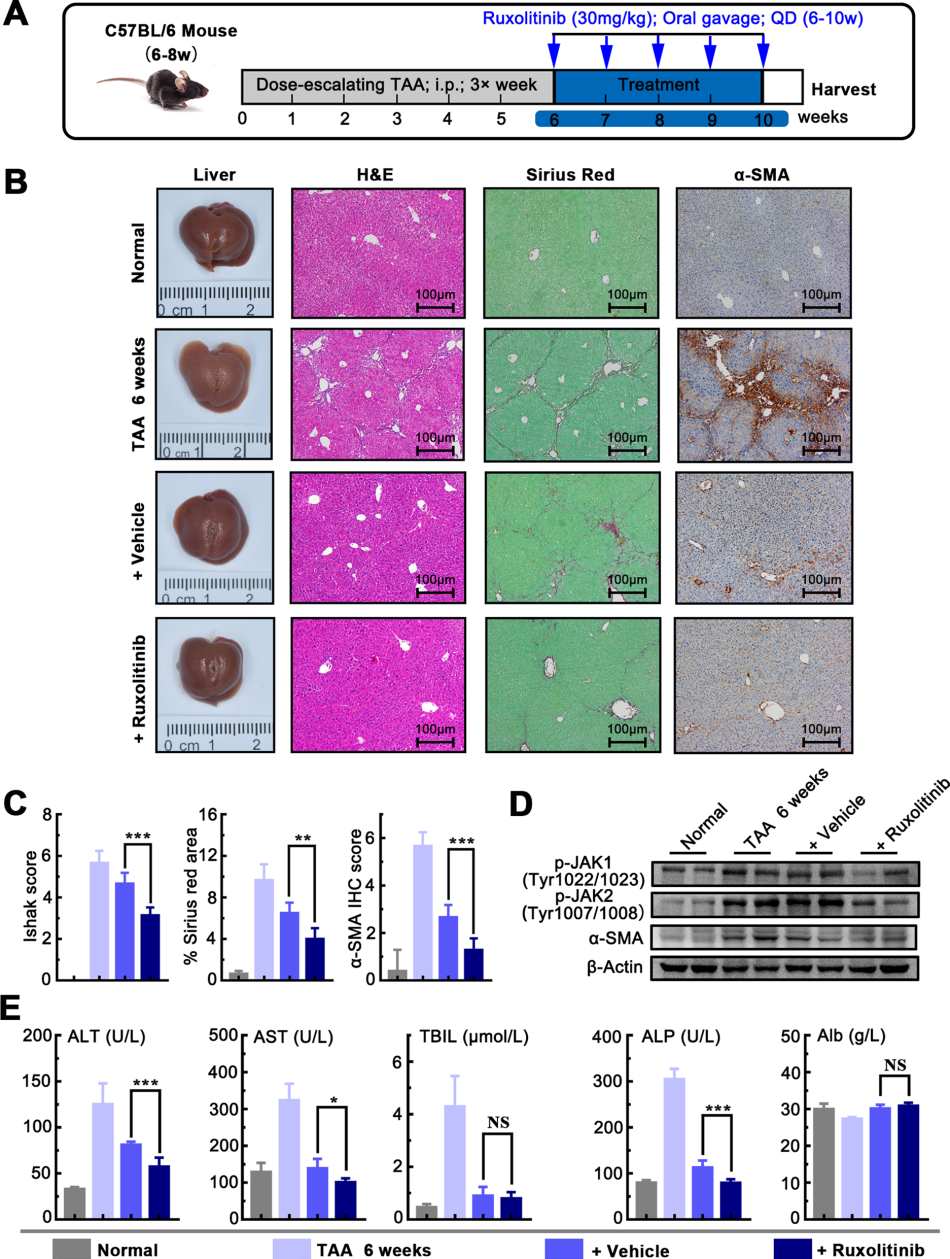
Ruxolitinib accelerates the reversal of liver fibrosis in the TAA mouse model. **A** Schematic of the experimental design of Ruxolitinib treatment in a TAA-induced fibrosis reversal model in mice. **B** Representative images of mouse livers stained with H&E, Sirius red and α-SMA antibodies. **C** Ruxolitinib reduced Ishak fibrosis score, and Sirius red, α-SMA IHC staining (× 100 magnification, scale bar = 100 μm). **D** Ruxolitinib reduced the protein expression of p-JAK1, p-JAK2, and α-SMA. **E** Serum levels of ALP, ALT, TBIL, ALP and Alb. Data presented are means ± SD. *NS* not significant; *P < 0.05; **P < 0.01; ***P < 0.001

**Fig.7:**
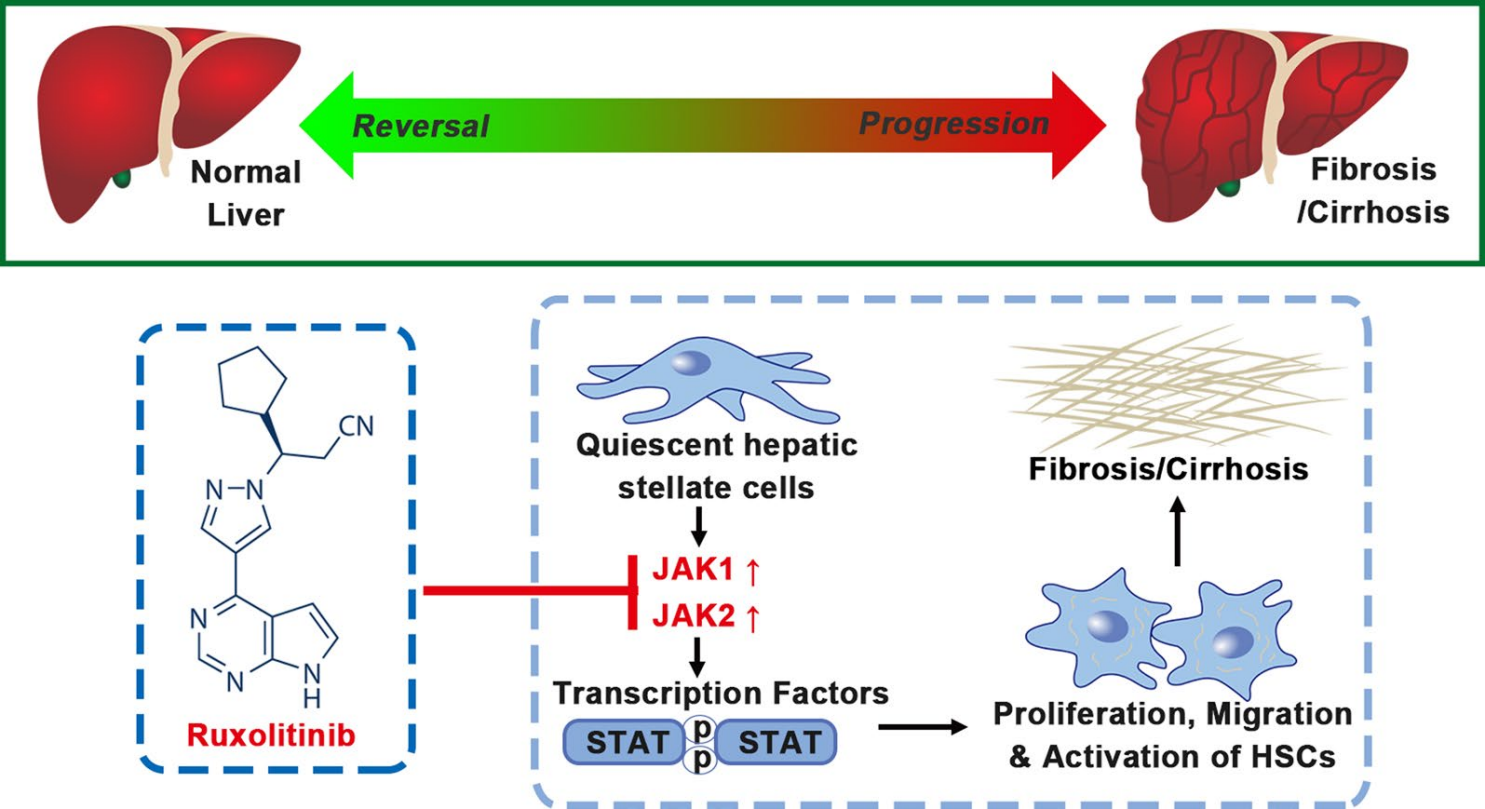
Proposed model of Ruxolitinib suppresses liver fibrosis progression and accelerates fibrosis reversal via selectively targeting JAK 1/2

To confirm the reversal effect of Ruxolitinib, another liver fibrosis mice model induced by TAA was used and consistent results were found. As shown in Fig. 6A, the mice were administered with TAA for 6 weeks, and then allowed to recover from 6 to 10 weeks, with or without treatment with Ruxolitinib. Similarly, Ruxolitinib was found to significantly reversed the degree of liver fibrosis and the activation of HSCs (Figs. 6B–D and 7) and decreased the levels of ALT, AST and ALP in 10 weeks mice. In conclusion, Ruxolitinib accelerates reversal of liver fibrosis and improve the liver damage in different mice model induced by CCl_4_ or TAA.

## Discussion

Liver fibrosis and cirrhosis are major health problems worldwide, causing more than 1 million deaths per year [30], for which there are currently no approved effective drugs [31]. Activated HSCs play a crucial role in liver fibrosis. However, the molecular mechanisms by which HSCs are activated and converted to a fibroblast phenotype are not fully understood. In this study, we found that enhanced JAK1 and JAK2 expression were associated with liver fibrosis/cirrhosis and liver cancer. IHC staining further demonstrated that the upregulation of JAK1 and JAK2 was mainly localized in hepatocytes and HSCs. More interestingly, we found that expression of JAK1 and JAK2 was positively correlated with the progression of liver cancer and the severity of liver fibrosis. Further, silencing of JAK1 and JAK2 down-regulated its downstream signaling and inhibited proliferation, activation, migration of HSCs. JAK1/2 inhibition had similar actions on HSCs in a concentration-dependent model in vitro and obviously attenuated the progress of liver fibrosis, promoted its reversal, and improved the liver damage in different liver fibrosis mice model induced by CCl_4_ or TAA. Therefore, JAK1/2 may be considered as a potential marker of activated HSCs and therapeutic targets in the treatment of liver fibrosis.

The JAK family is a class of non-receptor tyrosine kinases, play an important role in the development of many diseases, especially JAK2, which has been treated as a target of myeloproliferative diseases [32]. The existing reports suggests that STAT3 plays an important role in the development of liver fibrosis and demonstrates that STAT3 selective inhibitors can effectively attenuate the progression of liver fibrosis [18, 33–35]. However, JAK1 and JAK2, which are upstream of STAT3, have been mainly demonstrated to play a role in blood system diseases such as myelofibrosis and lymphoma [19, 20]. Interestingly, this study showed that JAK1/2 expression was up-regulated in liver fibrosis and HCC, and further positively associated with liver cancer progression and the severity of liver fibrosis, indicating that JAK1/2 may take an action in liver fibrosis. Activated HSCs have been demonstrated to play a central role in liver fibrogenesis by producing most of the ECM. HSCs are quiescent, and the underlying mechanism of activation is still not clear. We found We found JAK1/2 promoted the proliferation and activation of HSCs in vitro. Taken together, these results suggest that JAK1/2 promotes activation of HSCs and may be useful markers to monitor liver fibrosis and HCC development.

Subsequently evidence has demonstrated that Ruxolitinib had significant antifibrotic activity both in vitro and in vivo. Ruxolitinib is the most potent JAK1/2 inhibitor for blocking JAK1 phosphorylation at Tyr1022/1023 and JAK2 phosphorylation at Tyr1007/1008. A variety of chronic liver diseases lead to cirrhosis and HCC associated with high morbidity and mortality, while the treatments for advanced liver fibrosis and cirrhosis are still unsatisfactory. Therefore, understanding the mechanism and finding the effective drug to treat and reverse liver fibrosis are urgently needed [36]. Our date showed that Ruxolitinib significantly inhibited the activation of HSCs in vitro and suppressed liver fibrosis progression and accelerated reversal of liver fibrosis in independent murine models. We speculate that these phenomenon are mainly due to the anti-fibrotic and hepatoprotective effects of Ruxolitinib. However, the specific mechanism underlying these phenomenon are not fully elucidated. Further studies to explore the mechanism by primary HSCs and to investigate whether JAK1/2 inhibition prevents recurrence of HCC with the background of liver fibrosis in needed.

More interestingly, we observed that Ruxolitinib has a good effect on improving liver function in different liver fibrosis models, especially improving acute liver injury such as transaminase and bilirubin, which caused our concern. Patients with advanced cirrhosis and liver cancer miss effective treatment opportunities due to the accompanying liver function damage. Ruxolitinib has been shown to significantly improve acute liver injury, reduce transaminase and bilirubin, and achieve the purpose of improving liver function, which may win the opportunity for HCC patients to get anti-tumor treatments.

To date, there are no efficient anti-fibrotic therapies available for chronic liver disease and HCC. Innovative medical treatments to stop or even reverse fibrosis are urgently needed. Therefore, JAK1 and JAK2 might play a key role in the process of liver fibrogenesis, and its potential inhibitor, Ruxolitinib, may be clinically useful in preventing or treating liver fibrosis.

## Conclusions

In conclusion, JAK1/2 regulate the biological function of HSCs, highlighting its role in liver fibrosis and early prevention of HCC development and its inhibitor, Ruxolitinib, may be an effective drug for preventing and reversing liver fibrosis.

## Supplementary Information

**Additional file 1: Figure S1. **Ruxolitinib selectively inhibits JAK1 and JAK2 targets in LX-2 cells. **Figure S2.** The safety of Ruxolitinib in mice.

## Data Availability

The datasets generated and/or analyzed during the current study are not publicly available but are available from the corresponding author upon reasonable request.

## Abbreviations

Alb: Albumin
ALP: Alkaline phosphatase
ALT: Alanine aminotransferase
α-SMA: Alpha-Smooth muscle actin
AST: Aspartate aminotransferase
CCl_4_: Carbon tetrachloride
COL1A1: Collagen type I alpha 1
ECM: Extracellular matrix
FDA: Food and Drug Administration
HCC: Hepatocellular carcinoma
H&E: Hematoxylin–eosin
HSCs: Hepatic stellate cells
IC_50_: Half maximal inhibitory concentration
IHC: Immunohistochemistry
JAK: Janus kinase
JAK/STAT: Janus kinase/Signal Transducer and Activator of Transcription
NASH: Nonalcoholic steatohepatitis
PAI-1: Plasminogen activator Inhibitor-1
PDGFRβ: Platelet-derived growth factor receptor beta
siRNA: Small interfering RNA
SSc: Systemic sclerosis
TAA: Thioacetamide
TBIL: Total bilirubin
