# Supplementary material for: Ruxolitinib suppresses liver fibrosis progression and accelerates fibrosis reversal via selectively targeting Janus kinase 1/2
Source: J Transl Med. 2022 Apr 5;20:157. doi: 10.1186/s12967-022-03366-y (PMC8981941; doi:10.1186/s12967-022-03366-y)
Supplement: Supplementary file 1 — Additional file 1: Figure S1. Ruxolitinib selectively inhibits JAK1 and JAK2 targets in LX-2 cells. Figure S2. The safety of Ruxolitinib in mice. [file 12967_2022_3366_MOESM1_ESM.docx]

**Additional file 1: Figure S1. Ruxolitinib selectively inhibits JAK1 and JAK2 targets in LX-2 cells.** (A) The relative mRNA expression of JAK1 and JAK2 in LX-2 cells treated with different concentrations of Ruxolitinib. (B) The protein levels of JAK1, JAK2, p-JAK1 and p-JAK2 in LX-2 cells were treated with different concentrations of Ruxolitinib. Data presented are means ± SD. NS = not significant.

**Additional file 1: Figure S2. The safety of Ruxolitinib in mice.** (A) Representative images of mouse livers stained with H&E (× 100 magnification, scale bar = 100μm). (B) Serum levels of ALP, ALT, TBIL, ALP and Alb. Data presented are means ± SD. NS = not significant.


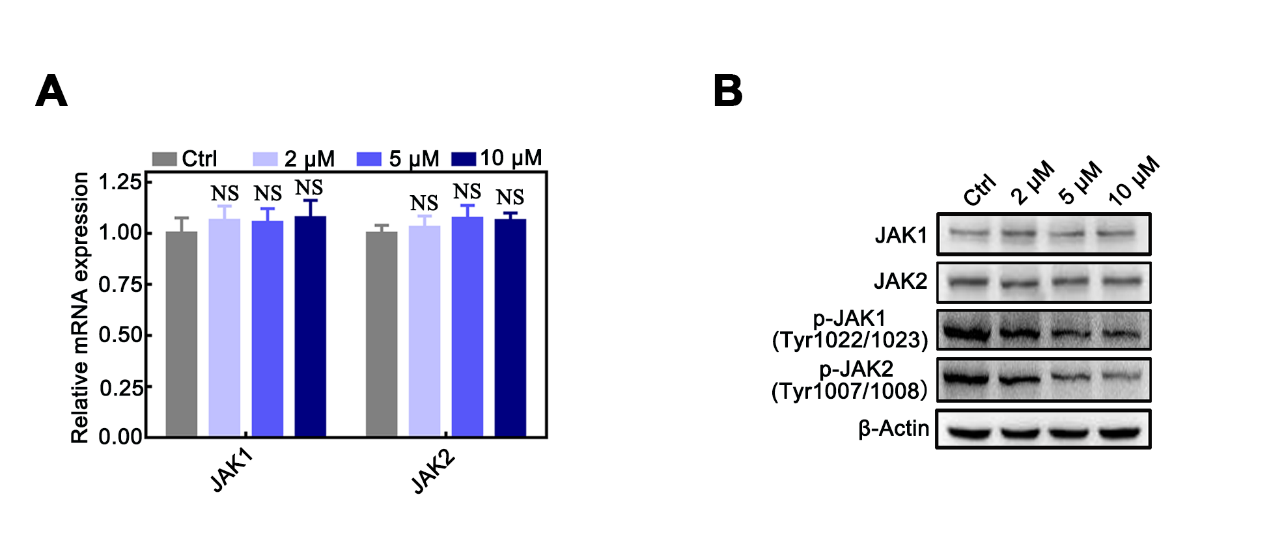


**Additional file 1: Figure S1. Ruxolitinib selectively inhibits JAK1 and JAK2 targets in LX-2 cells.**


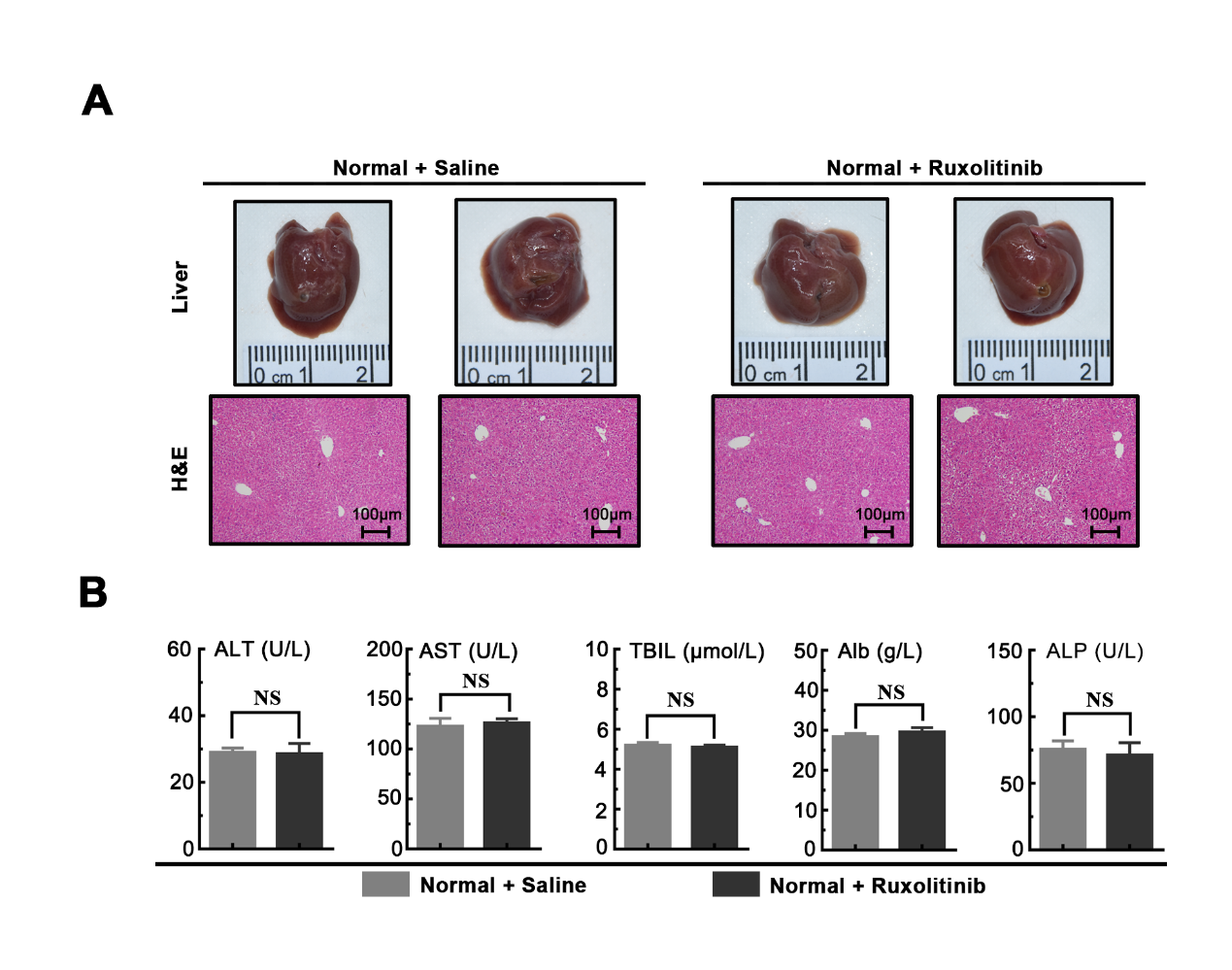


**Additional file 1: Figure S2. The safety of Ruxolitinib in mice.**
